# ‘Fostering critical engagement rather than blind devotion’: calling for transdisciplinary microbiology research

**DOI:** 10.1111/1462-2920.15801

**Published:** 2021-10-10

**Authors:** S. B. Barendse

**Affiliations:** ^1^ Beta Faculty Vrije Universiteit Amsterdam Amsterdam The Netherlands

This highlight is dedicated to Bradshaw's call for interdisciplinary research in this issue, i.e. stressing the importance of microbiological literacy in the current zeitgeist and the contribution that collaboration between microbiologists and social scientists can make to this. I, as social scientist and science communication researcher, want to take his call one step further by arguing that the examples he illustrates are also examples of transdisciplinary research, and argue that this approach can make a contribution to the field of microbiological research.

Transdisciplinary research, i.e. scientific inquiry that cuts across disciplines ‘to overcome the mismatch between knowledge production in academia, on the one hand, and knowledge requests for solving societal problems, on the other' (Hadorn *et al.,* 2008, p.4) understands that if we want to solve complex societal problems, these problems should be accounted for in collaboration across, but also transcend scientific disciplinaries. Take for example the quick emergence of the COVID‐19 pandemic, which has its roots in microbiology, but impacted politics, science, the environment and society and is dealt with in all of these domains, making it a *socio*scientific issue. Citizens have often felt left out in the decision‐making process surrounding COVID‐19 (e.g. before in safety measures, now in vaccine passports) which led to protests and incomprehension between various layers within society. More than enough information was communicated about the virus and affiliated measures by the government and science, but people felt misunderstood and unheard: a communication mismatch. In this abundance of COVID‐19 information, how come so many people have attained a different perspective on COVID‐19?

A frequently heard explanation for the mismatch is society's lack of microbial/scientific literacy. It is evident that society's understanding of microbes (*microbial literacy*) to make informed decisions regarding vaccination and safety measures and to be able to critically assess discourse in these areas has played a role here. Timmis *et al*. (2019) previously reported on the urgent need for microbial literacy in society, especially in relation to the emergence of such pandemics. However, how citizens make sense of COVID‐19 on the micro‐level (i.e. everyday conversations and experiences) often relies on emotions and feelings they acquire in conversations with acquaintances rather than on rational, factual communication by governmental instances (Rerimassie *et al*., 2021). Apparently, there is a whole world where individual *sensemaking* takes place and where scientific, evidence‐based knowledge seeps in only to a very limited extent, especially in hard‐to‐reach audiences with low literacy, such as local and rural communities, and individuals that are not interested in science topics (Milani *et al*., 2021). There again we see this mismatch appearing: a discrepancy between science's information dissemination practices and people's sensemaking of science‐related phenomena and events. How science and policymakers address society has not had the desired effect, making some of the current communication channels inadequate.

To explain this mismatch, we can take on a social scientist's perspective, where we focus on the term *literacy*. Literacy has long been scrutinized in the field of science communication and science–technology–society studies (Liu, 2009; Sismondo, 2010; Valladares, 2021). *Literacy* is often associated with the concept of *deficit‐thinking*: the persistent view within science that assumes that society's mistrust towards science is grounded in a *deficit* of citizens' scientific knowledge, and society is therefore responsible for conflicts and misunderstandings between science and society. *If they would only understand science better*, *they would be less opposing to scientific innovations*. To overcome this knowledge deficit, it has been thought that scientists should better inform and educate society, therewith restoring society's trust in science. This has been a common approach for decades and even though science literacy in society has slowly increased over this period of time, there still are large variations in literacy within society (e.g. National Academies of Science, Engineering and Medicine, 2016), indicating that there are illiterate publics that are not reached by our information and education efforts.

Another rationale in deficit‐thinking is that citizens let emotions and values interfere in how they take up information, which would make citizens by definition unfit to come up with decisions in complex issues that require rationality well‐informed choices. Academic training, conversely, should enable scientists to exclude emotions and values in our work and help provide untainted evidence‐based information that is required to participate in debates about socioscientific issues and decision‐making. This belief, the belief that the public is emotional and ignorant whilst science is rational and reliable, is still common in scientists nowadays (Barendse *et al*., 2021). Such deficit‐thinking views demarcate science from society in a problematic way and disregard the previously mentioned sensemaking practices of citizens, eventually contributing to polarization between science and society.

I, therefore, applaud the views of Timmis *et al*. (2019), who look at the term *literacy* from a different perspective: they follow the belief that broad and freely available education enables citizens to become literate and correctly inform themselves and others, but, more importantly, should empower citizens to participate in debates and deliberation. ‘*The goal of all science education or science communication research should be to foster critical engagement rather than blind devotion*’ (Baram‐Tsabari and Osborne, 2015, p. 138) – to reduce the epistemic distance between scientists and lay people, we should attempt to engage the public in dialogue rather than focus on dissemination of content knowledge. This is one of the key pillars of transdisciplinary research: equally valuing scientific knowledge and experiential knowledge. If we recognize the *socio* in socioscientific issues, the citizen suddenly becomes an expert. This creates support, trust and equality, and highlights important aspects to a research subject that researchers would not have thought of themselves.

Then the question remains what engaged publics in the dialogue surrounding socioscientific issues implies for microbiology research. In this issue, Bradshaw provides the example of Lorimer *et al*. (2019)'s *participatory genomics*, illustrating how new knowledge about hygiene practices in the composition of the domestic microbiome was co‐created by microbiologists in collaboration with citizen participants via collaborative research design, experimentation and result interpretation – a symbiose of microbiologists' knowledge and experiential knowledge. Citizens are *consults* in this example and are only involved in a small part of the research process, that of data collection. The other example of Bradshaw (*political cyanobacteria*; Waterton and Tsouvalis, 2015) takes transdisciplinarity one step further: in the local issue of a lake polluted by algae, microbiologists, social scientists, citizens, farmers and other stakeholders were involved in multiple steps of research and subsequent decision‐making. Opening up this issue to a broader set of stakeholders shed new light on the potential origins of the algae pollution would not have occurred mono‐disciplinary researchers (*substantive reasoning*), while simultaneously creating support and trust within and between everyone involved.

These are prime examples of the benefits of transdisciplinary research with microbiologists, but transdisciplinary research also has applications that go beyond laboratory research and solving local problems. Scientific knowledge and experiential knowledge can also be used to collaboratively determine desired futures for science and society. Citizens must then be involved in the process from the outset, because *‘if we want to develop applications robustly and in the public interest*, *it is important to organize reflexive strategies of assessment and engagement in early stages of development*.’ (Betten *et al*., 2018, p. 21). For instance, in a recent joint‐project by Betten *et al*. (2018) and Stemerding *et al*. (2019), synthetic biology researchers and societal stakeholders came together to co‐create future scenarios about antibiotic resistance and renewable energy. The aim was to create a transdisciplinary guideline for future innovations in both fields, taking into account both technological options and societal objectives. Their concept of *building future scenarios as a tool for responsible research and innovation* consisted of two trajectories. The researchers followed a technological options‐oriented approach to explore the plausibility and feasibility of innovations that might be involved in the future of antibiotic resistance and renewable energy. Societal stakeholders, on the other hand, followed a societal objectives‐oriented approach and examined the nature of such future scenarios as well by exploring the needs, values and purposes of the societal challenge and the potential role of synthetic biology herein. Finally, both parties were asked to share their findings in workshops – ‘*a dialogue in which connections are made between societal goals and technology’* (Stemerding *et al*., 2019, p. 219) – and came to a mutual understanding regarding these possible futures.

The approach of constructing future scenarios in a transdisciplinary fashion is not only limited to research but also already has application within public health, for example with the One Health approach that was started with the WHO. I argue that this approach is applicable to all microbiology‐related crises as described in Timmis *et al*. (2019): the antibiotic resistance crisis; the return of virtually eradicated childhood diseases; the rise of allergies; the greenhouse gas crisis; the soil crisis; and the pollutant accumulation in the environment and food web. Not only literacy is important to collaboratively solve these crises but also the experience of society is important and in similar fashion are these issues not just scientific‐microbial issues but socioscientific issues. The responsibility, therefore, lies with all of those involved (public, scientists, policymakers) for both applying transdisciplinary approaches as well as solving of the crises that threaten our existence.
